# The dynamics of cell death patterns and regeneration during acute liver injury in mice

**DOI:** 10.1002/2211-5463.13383

**Published:** 2022-03-05

**Authors:** Shuai Shao, Yu Zhang, Guantong Li, Zhenjun Yu, Yingying Cao, Lina Zheng, Kun Zhang, Xiaohui Han, Zhemin Shi, Hongmei Cui, Xiaomeng Song, Wei Hong, Tao Han

**Affiliations:** ^1^ The School of Medicine NanKai University Tianjin China; ^2^ Department of Hepatology and Gastroenterology The Third Central Clinical College of Tianjin Medical University China; ^3^ 12610 Department of Histology and Embryology School of Basic Medical Sciences Tianjin Medical University China; ^4^ Department of Hepatology and Gastroenterology Tianjin Union Medical Center Nankai University China; ^5^ Department of Hepatology and Gastroenterology Tianjin Third Central Hospital affiliated to Nankai University China

**Keywords:** acute liver injury, cell death, cytokines, hepatocyte, prognosis, regeneration

## Abstract

Acute liver injury is a serious clinical syndrome with multiple causes and unclear pathological process. Here, CCl_4_‐ and D‐galactosamine/lipopolysaccharide (D‐gal/LPS)‐induced acute liver injury was established to explore the cell death patterns and determine whether or not liver regeneration occurred. In CCl_4_‐induced hepatic injury, three phases, including the early, progressive, and recovery phase, were considered based on alterations of serum transaminases and liver morphology. Moreover, in this model, cytokines exhibited double‐peak fluctuations; apoptosis and pyroptosis persisted throughout all phases; autophagy occurred in the early and the progressive phases; and sufficient and timely hepatocyte regeneration was observed only during the recovery phase. All of these phenomena contribute to mild liver injury and subsequent regeneration. Strikingly, only the early and progressive phases were observed in the D‐gal/LPS model. Slight pyroptosis occurred in the early phase but diminished in the progressive phase, while apoptosis, reduced autophagy, and slight but subsequently diminished regeneration occurred only during the progressive phase, accompanied by a strong cytokine storm, resulting in severe liver injury with high mortality. Taken together, our work reveals variable modes and dynamics of cell death and regeneration, which lead to different consequences for mild and severe acute liver injury, providing a helpful reference for clinical therapy and prognosis.

## Graphical abstract

In this study, we detected different modes of cell death during acute liver injury in a mouse model. We revealed that variable modes and dynamics of cell death and regeneration lead to different consequences for mild and severe acute liver injury, providing a helpful reference for clinical therapy and prognosis.

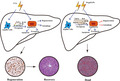

**Abbreviations**
ALT: alanine aminotransferase
AST: aspartate aminotransferase
CCl_4_
: carbon tetrachloride
D‐gal/LPS: d‐galactosamine/lipopolysaccharide
H&E: hematoxylin and eosin
IHC: immunohistochemistry
LDH: lactate dehydrogenase
qRT‐PCR: quantitative real‐time PCR
TUNEL: terminal deoxynucleotidyl transferase dUTP nick end labeling

Acute liver injury is defined as liver dysfunction with various etiologies including drugs and poison, virial hepatitis, ischemia, or other causes. Severe liver injury can often lead to acute liver failure, which is a life‐threatening disorder contributed to dramatically increased morbidity and mortality [1]. Liver transplantation is currently the most efficient therapy for liver failure. However, it is limited by the scarcity of donor, expensive medical cost, and surgical risk and requires lifelong immunosuppressant agents. Therefore, prevention and treatment of acute liver injury or failure remains a worldwide challenge.

Under normal conditions, a balance exists between cell death and regeneration in the liver, and the remaining healthy liver has a strong regenerative capacity when damaged by external stimuli. However, liver failure or even death can occur if regeneration and repair of hepatocytes is insufficient when damage continued to progress [2]. Cell death plays an important role in the development of liver disease. In addition to apoptosis and necrosis, pyroptosis, autophagy, necroptosis, ferroptosis, and other modes of death have been increasingly studied in liver disease in recent years [3].

Pyroptosis, a novel programmed and proinflammatory cell death initiated by inflammasomes and induced by gasdermin D (GSDMD), leads to pore formation in the plasma membrane, fluid influx, cell swelling, rapid plasma membrane rupture, and massive release of proinflammatory cytokines, such as interleukin‐1β (IL‐1β) and IL‐18, causing an inflammatory response [4, 5]. Pyroptosis has been increasingly studied in acute injury, hepatitis, alcoholic liver disease, and nonalcoholic fatty liver disease [6, 7, 8]. It has been reported that nucleotide‐binding and oligomerization domain‐like receptor (NLR) family pyrin domain containing 3(NLRP3), caspase‐1, and gasdermin D (GSDMD) are activated in D‐galactosamine/lipopolysaccharide (D‐gal/LPS)‐induced acute liver failure [9]. A study indicated that dihydromyricetin ameliorates carbon tetrachloride (CCl_4_)‐induced chronic liver injury by reducing pyroptosis [10]. Autophagy is an evolutionarily conserved degradation pathways for cytosolic macromolecules and damaged/excess organelles, which has been known to exhibit a pivotal role in maintaining cellular homeostasis [11]. When autophagy is induced, cytosolic components are encapsulated in a phagophore or autophagosome. The autophagosome fuses with a lysosome, allowing degradation of encapsulated products, whereby molecules and proteins may be recycled to improve cell survival [12, 13]. It has been suggested that autophagy not only modulates normal liver function but also participates in the pathogenesis of various liver disorders. Indeed, it has been reported that autophagy protects against acetaminophen‐induced liver damage in mice by the removal of acetaminophen protein adducts [14, 15, 16]. The selective loss of endothelial autophagy impairs liver sinusoidal endothelial cell’s ability to handle oxidative stress and aggravates CCl_4_‐induced fibrosis [17].

Liver regeneration is a compensatory process that replaces functional liver mass lost caused by injuries or diseases. Liver regeneration has been well described for several toxicants, such as CCl_4_, acetaminophen, and chloroform [18]. The findings from acute studies suggest that liver regeneration plays a crucial role in determining the final outcome of toxicant‐induced acute liver injury, such that timely and proportionate stimulation of regeneration leads to regression of injury, but delayed or inhibited regeneration culminates in the progression of injury and death [19]. However, the dynamics of cell death patterns and regeneration in the development of acute liver injury have not been systematically studied.

In this study, CCl_4_ and D‐gal/LPS were used to construct two acute liver injury models with differing severities and prognoses, and explore the dynamic evolution of multiple cell death patterns and cell regeneration in different acute liver injury models. We found that the severe D‐gal/LPS liver injury model showed significant apoptosis and suppressed autophagy and regeneration, while the mild CCl_4_ liver injury model showed active autophagy and timely and sufficient hepatocyte regeneration; and there were significant differences in the dynamic evolution of cell death and regeneration between the two models, which were closely related to the prognosis.

## Materials and methods

### Animal model and treatment

Animal protocols were approved by the Tianjin Third Central Hospital Animal Care and Use Committee. The methods were carried out in accordance with the approved guidelines. Eight‐week‐old BALB/c and C57BL/6J male mice were obtained from the Institute of Laboratory Animal Sciences, Chinese Academy of Medical Sciences (CAMS), and Peking Union Medical College (PUMC; Beijing, China). Mice were maintained in a 12‐h light/dark cycle at 22–25 °C with free access to food and water. After acclimatization for 1 week, the mouse liver injury model was induced by an injection of CCl_4_ (Sigma‐Aldrich, St. Louis, MO, USA) or D‐gal/LPS (Sigma‐Aldrich). For the CCl_4_‐induced mouse liver injury model, 48 BALB/c mice were randomly divided into eight groups as follows: control (*n* = 6), 6‐h liver injury (*n* = 6), 12‐h liver injury (*n* = 6), 24‐h liver injury (*n* = 6), 48‐h liver injury (*n* = 6), 72‐h liver injury (*n* = 6), 96‐h liver injury (*n* = 6), and 8‐day liver injury (*n* = 6). 10% CCl_4_ (v/v) dissolved in olive oil (1 mL·kg^−1^ body weight) was administered to all CCl_4_ groups via intraperitoneal injection, whereas control mice were administered an equivalent volume of olive oil. For the D‐gal/LPS‐induced mouse liver injury model, mice received an intraperitoneal injection of D‐gal and LPS (700 mg·kg^−1^ and 100 μg·kg^−1^, respectively, dissolved in normal saline), whereas controls were administered an equivalent volume of normal saline. We observed that mice died between 6 and 7 h after administering D‐gal/LPS. Finally, 40 C57BL/6J mice were randomly assigned to four groups as follows: control (*n* = 8), 2‐h liver injury (*n* = 8), 4‐h liver injury (*n* = 8), and 6‐h liver injury (*n* = 16). All mice were euthanized with 3% sodium pentobarbital (45 mg·kg^−1^, intraperitoneal injection). Liver specimens and blood samples were subsequently obtained for analyses.

### Isolation and culture of primary hepatocytes and KCs

Primary mouse hepatocytes were isolated in situ and perfused sequentially with 30 mL SC1 solution and 30 mL 0.05% collagenase IV solution. Next, hepatocytes were centrifuged 3× at 50 ***g*** for 4 min to obtain a pellet. The primary mouse Kupffer cells (KCs) were isolated by pronase/collagenase perfusion digestion followed by subsequent density gradient centrifugation, as previously described. Cell viability was determined on the pelleted cells using the trypan blue exclusion method.

### Measurement of serum biochemical markers

Blood samples were collected from euthanized mice, and serum levels of aspartate aminotransferase (AST), alanine aminotransferase (ALT), and lactate dehydrogenase (LDH) were measured using commercially available diagnostic kits (Nanjing Jiancheng Bioengineering Institute, Nanjing, China). The final data were presented as units·liter^−1^ (U·L^−1^).

### Histology and immunohistochemistry

Hematoxylin and eosin (H&E) staining was used for histological examination of tissue samples. Briefly, specimens were fixed in 10% formalin for 2 days, subjected to an ethanol gradient, and embedded in paraffin. Thin sections (5 μm) were stained with hematoxylin for 1–2 min at room temperature. After washing, slices were stained with eosin for 30–60 s at room temperature. Immunohistochemistry (IHC) was performed as described previously [20]. Images under 400× magnification were randomly selected and captured using a light microscope.

### TUNEL assay

An *in situ* cell detection kit (Roche, Basel, Switzerland) was used for terminal deoxynucleotidyl transferase dUTP nick end labeling (TUNEL) staining according to the manufacturer’s protocol. After dewaxing and rehydration, we pretreated tissue sections with 3% H_2_O_2_ and subsequent proteinase K permeation. Pretreatment with DNase I served as a positive control, and TUNEL reaction mixture lacking terminal transferase (TdT) was used as a negative control. Samples were analyzed using light microscopy (400× magnification).

### Western blot analysis

Immunoblotting analysis was performed as described previously [21] using the following primary antibodies: caspase‐3 (1 : 1000; Cell Signaling Technology Inc., Danvers, MA, USA), BCL2‐associated X (BAX; 1 : 1000; Abcam, Cambridge, UK), caspase‐1 (1 : 1000; Abcam), caspase‐11 (1 : 1000; Abcam), GSDMD (1 : 1000; Abcam), NLRP3 (1 : 1000; Cell Signaling Technology Inc.), LC3‐B (1 : 1000; Cell Signaling Technology Inc.), mixed lineage kinase domain‐like protein (MLKL; 1 : 1000; Cell Signaling Technology Inc.), glutathione peroxidase 4 (GPX4; 1 : 1000; Cell Signaling Technology Inc.), proliferating cell nuclear antigen (PCNA; 1 : 1000; Cell Signaling Technology Inc.), and cyclin D1 (1 : 1000; Abcam). GAPDH was used as an internal control.

### Quantitative real‐time PCR

Quantitative real‐time PCR (qRT‐PCR) analysis was performed as described previously [20]. The expression level of housekeeping gene GAPDH was used to normalize the expression level of the genes of interest. Primer sequences for mouse genes were *Gapdh* sense 5′‐GGCATGGACTGTGGTCATGAG‐3′ and antisense 5′‐TGCACCACCAACTGCTTAGC‐3′; tumor necrosis factor‐α (*Tnf‐α*) sense 5′‐CATCTTCTCAAAATTCGAGTGACAA‐3′ and antisense 5′‐TGGGAGTAGACA‐AGGTACAACCC‐3′; monocyte chemoattractant protein‐1 (*Mcp‐1*) sense 5′‐GTTAACGCCCCACTCACCTG‐3′ and antisense 5′‐GGGCCGGGGTATGTAACTCA‐3′; *Il‐6* sense 5′‐AGTTGCCTTCTTGGGACTGA‐3′ and antisense 5′‐TCCACGATTTCCCAGAGAAC‐3′; *Il‐10* sense 5′‐GCTCTTGCACTACCAAAGCC‐3′ and antisense 5′‐CTGCTGATCCTCATGCCAGT‐3′; *Il‐1β* sense 5′‐GTCGCTCAGGGTCACAAGAA‐3′ and antisense 5′‐GTGCTGCCTAATGTCCCCTT‐3′; and *Pcna* sense 5′‐TTTGAGGCACGCCTGATCC‐3′ and antisense 5′‐GGAGACGTGAGACGAGTCCAT‐3′.

### Statistical analyses

Statistical analysis was performed using the spss 19.0 (SPSS for Windows, Chicago, IL, USA) software. The data were expressed as mean ± SEM (standard error of the mean). Statistical analyses were performed using either Student’s *t*‐test (two‐group comparison) or one‐way analysis of variance (more than two groups), followed by post hoc comparison. Differences with *P* < 0.05 were considered significant.

## Results

### CCl4‐ and D‐gal/LPS‐induced acute liver injury in mice

**Fig. 1:**
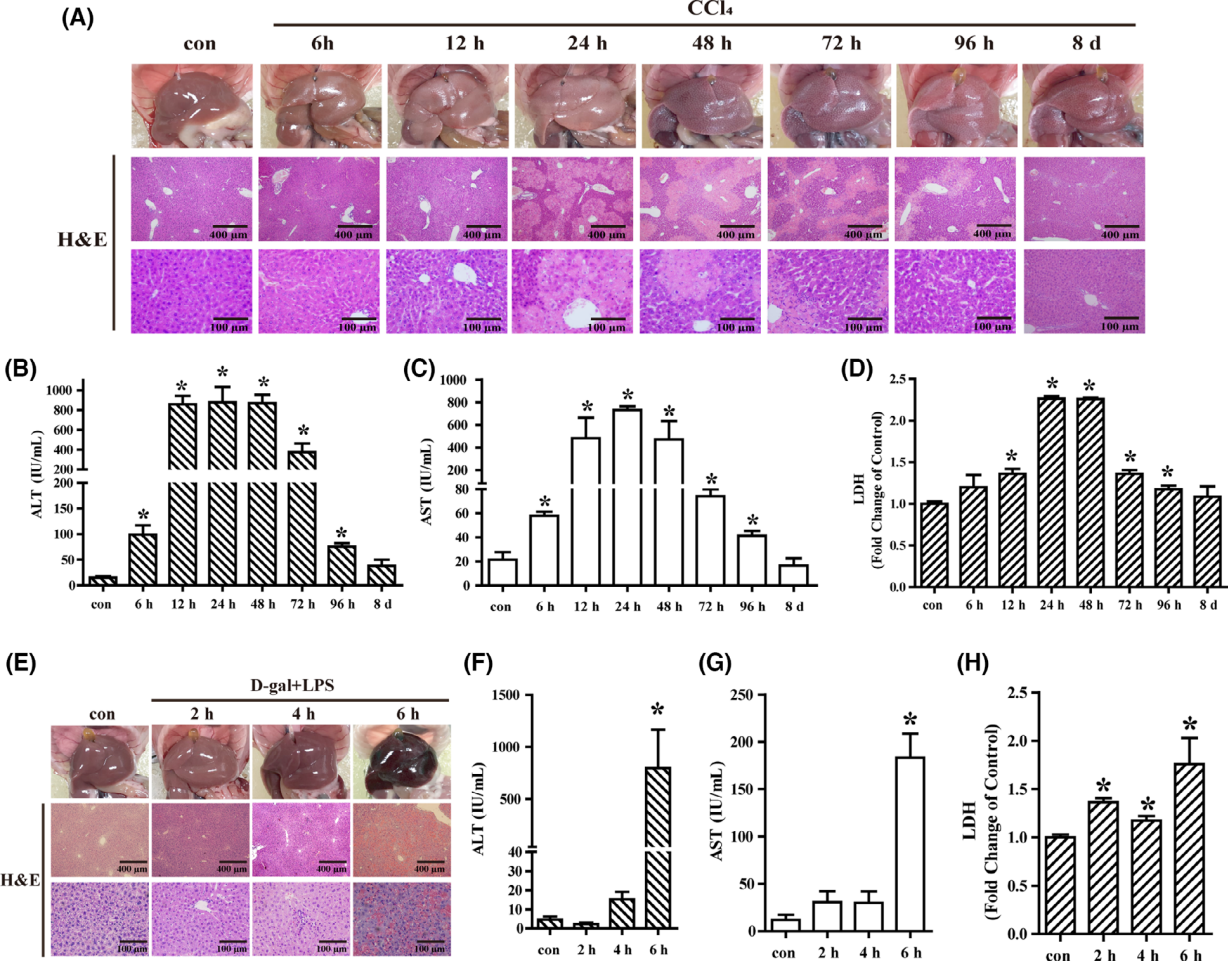
CCl_4_‐ and D‐gal/LPS‐induced acute liver injury models. Mice were injected with either CCl_4_ (olive oil as control) or D‐gal/LPS (saline as control) to induce acute liver injury and were sacrificed at different time points. Macroscopic appearance and H&E staining of the liver tissues for the CCl_4_ (A)‐ and D‐gal/LPS (E)‐treated mice; serum ALT (B, F), AST (C, G), and LDH (D, H) were measured. Scale bar, 400 μm for 10× and 100 μm for 40×. The data were expressed as the mean ± SEM for at least triplicate experiments. *P* values were analyzed by Student’s *t*‐test. *P < 0.05 vs control.

To establish liver injury animal models, the mice were injected with either CCl_4_ or D‐gal/LPS intraperitoneally. The degree of liver injury was evaluated by macroscopic examination, H&E staining, and measurement of AST, ALT, and LDH. As shown in Fig. 1A–D, the CCl_4_‐injected livers were enlarged with some bleeding surface macroscopically. H&E staining demonstrated a submassive centrilobular necrosis, reaching the most severity at 24 h when the serum ALT, AST, and LDH showed peak levels as well, compared with the control, while the D‐gal/LPS‐injured mice showed a dark red and slightly roughened surface, irregularly distributed hepatocytes, and massive cell death as revealed by H&E staining, and a peak level of serum ALT, AST, and LDH at 6 h (Fig. 1E–H). Notably, since all the D‐gal/LPS‐injured mice died between 6 and 7 h, the detection of cytokines and patterns of cell death was up to 6 h. Taken together, the results showed that the acute liver injury models were successfully constructed.

### Expression profiles of cytokines in CCl4‐induced liver injury

**Fig. 2:**
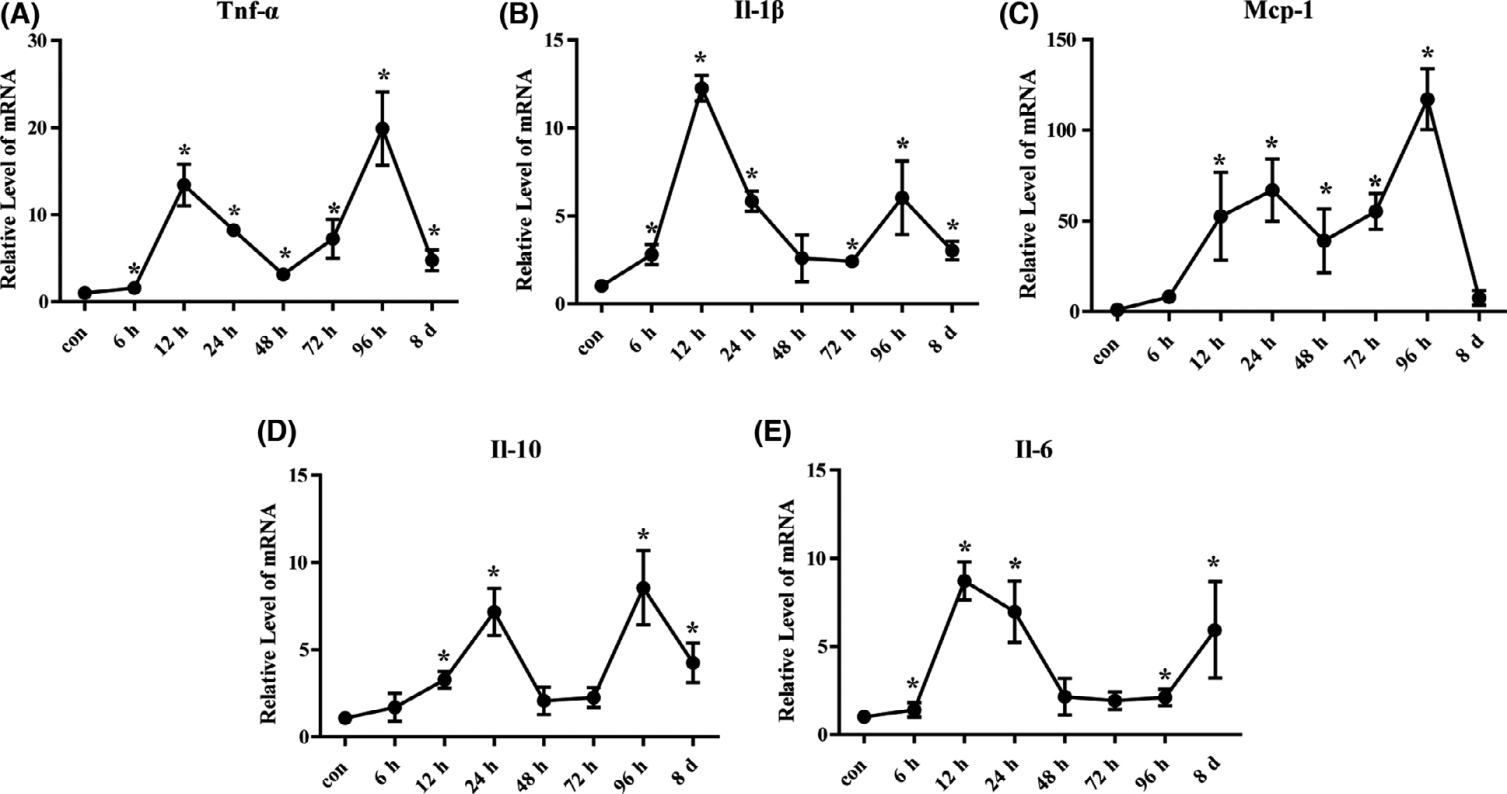
Cytokine expression in CCl_4_‐induced acute liver injury model mice. The expression of inflammation‐related genes TNF‐α (A), IL‐1β (B), Mcp‐1 (C), IL‐10 (D), and IL‐6 (E) was evaluated by qRT‐PCR. The data were expressed as the mean ± SEM for at least triplicate. *P* values were analyzed by Student’s *t*‐test. *P < 0.05 vs control.

To investigate the inflammatory response associated with pathogenesis and an important feature of acute liver injury, the expression of cytokines in the liver was detected. The results of qRT‐PCR revealed that the level of cytokines was enhanced at 6 h poststimulation and TNF‐α, IL‐1β, and IL‐6 reached a peak at 12 h, while the peak of Mcp‐1 and IL‐10 was at 24 h, suggesting the cytokines induce hepatic injury. After a gradual decrease, the level of cytokines increased to another peak at 96 h, correlated with the decreased level of enzymes (Fig. 1B–D), indicating this peak may be associated with liver recovery (Fig. 2A–E). Thus, cytokines were elevated and dynamically changed after CCl_4_‐induced liver injury.

### Dynamic changes in cell death patterns in CCl4‐induced liver injury

**Fig. 3:**
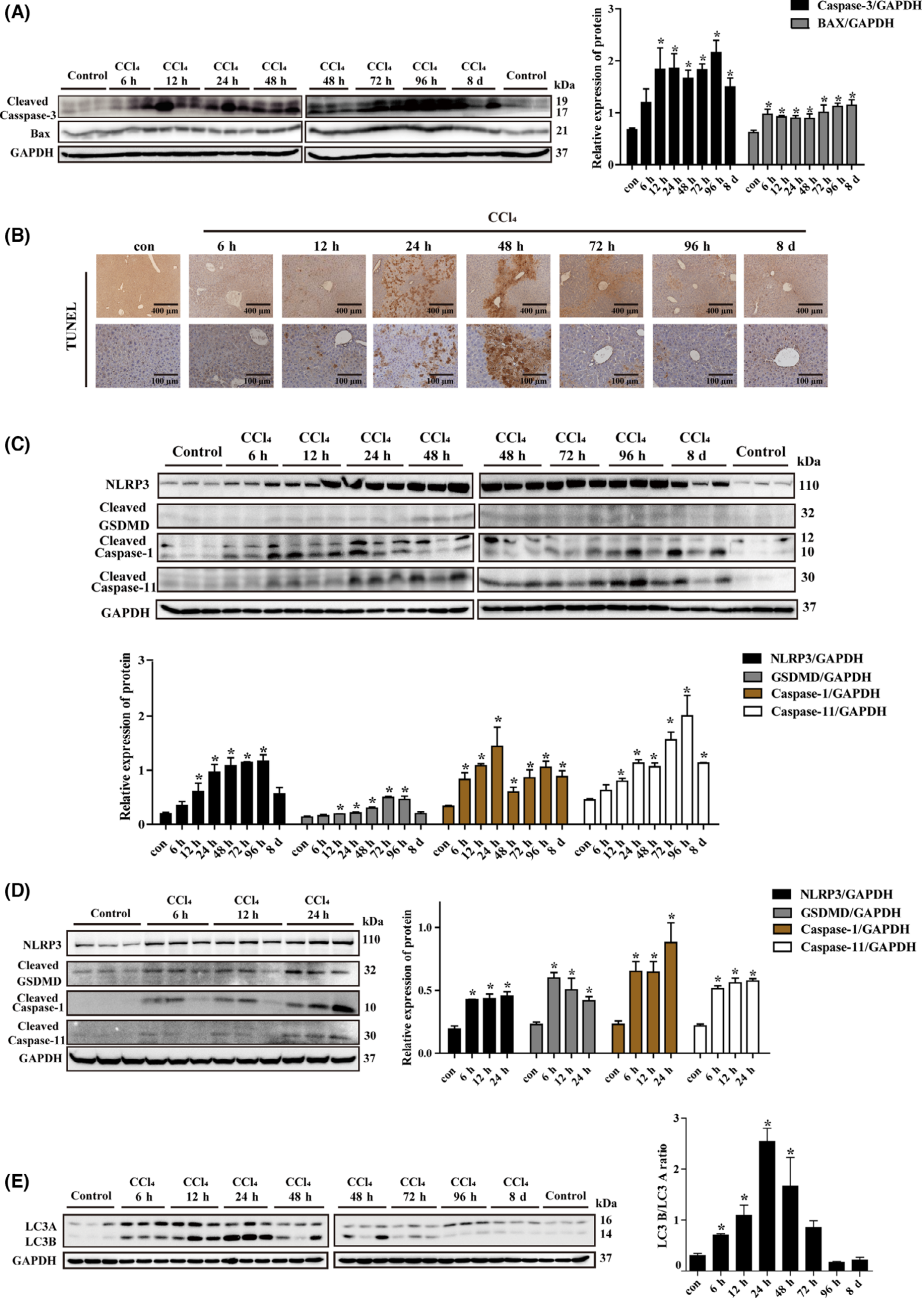
Expression of apoptosis‐, pyroptosis‐, and autophagy‐related genes in liver tissues of CCl_4_‐induced acute liver injury model mice. (A) The protein level of apoptosis genes in liver tissues was determined by western blot; (B) TUNEL detection of apoptotic gene expression in liver tissue; the protein level of pyroptosis genes in liver tissue (C) and KCs (D) determined by western blot; and (E) the protein level of autophagy‐related gene in liver tissue determined by western blot. GAPDH was used as an internal control. Scale bar, 400 μm for 10× and 100 μm for 40×. The data were expressed as the mean ± SEM for at least triplicate experiments. *P* values were analyzed by Student’s *t*‐test. *P < 0.05 vs control.

In order to understand the potential involvement of different modes of death in liver injury, we firstly determined apoptosis‐associated genes at different stages. The data showed that, compared with the control, the protein level of cleaved caspase‐3 and BAX was increased from 6 h until 8 days (Fig. 3A). In addition, TUNEL staining showed apoptosis occurred at 6 h and continued to 8 days (Fig. 3B). Taken together, our results indicated that apoptosis was associated with liver injury and recovery.

Next, we investigated whether pyroptosis is involved in liver injury by determination of the relevant markers. The results of western blot revealed that the protein level of NLRP3 and cleaved caspase‐1 was increased at 6 h, while that of GSDMD‐N and cleaved caspase‐11 was at 12 h, and the enhancement was remained until 8 d in the liver tissue (Fig. 3C), indicating that pyroptosis is throughout the process of liver injury. To identify the cells in which pyroptosis occurs, we isolated primary hepatocytes and KCs from the injured liver and detected the expression of pyroptosis‐associated genes. The results demonstrated that the protein level of NLRP3 and cleaved caspase‐1 was increased at 24 h, while that of GSDMD‐N and cleaved caspase‐11 was increased at 48h in hepatocytes. However, the expression of all genes was increased at 6 h in KCs (Fig. 3D, Fig. S1A), showing that pyroptosis in KCs occurred in the early stage of liver injury.

It has been known that organ injury involves autophagy [22]. To elucidate the potential role of autophagy in liver injury, the expression of autophagy‐associated proteins, the cytosolic LC3‐A and the lipid‐bound form LC3‐B, was determined. The data showed that LC3‐A was weakly expressed in the normal liver while LC3‐B was nearly not detectable. Since LC3‐A is converted to LC3‐B when autophagy occurs, the ratio of LC3‐B to LC3‐A began to increase at 6 h after injury and peaked at 24 h, and resumed to normal level at 72 h, suggesting that autophagy may possibly serve as a compensatory mechanism during early liver injury (Fig. 3E).

In addition, the expression of MLKL and GPX4, which are necroptosis‐ and ferroptosis‐associated proteins, respectively, showed no significant alteration in the progression of CCl_4_‐induced acute liver injury (Fig. S1B).

### Dynamics of liver regeneration in CCl4‐induced liver injury

**Fig. 4:**
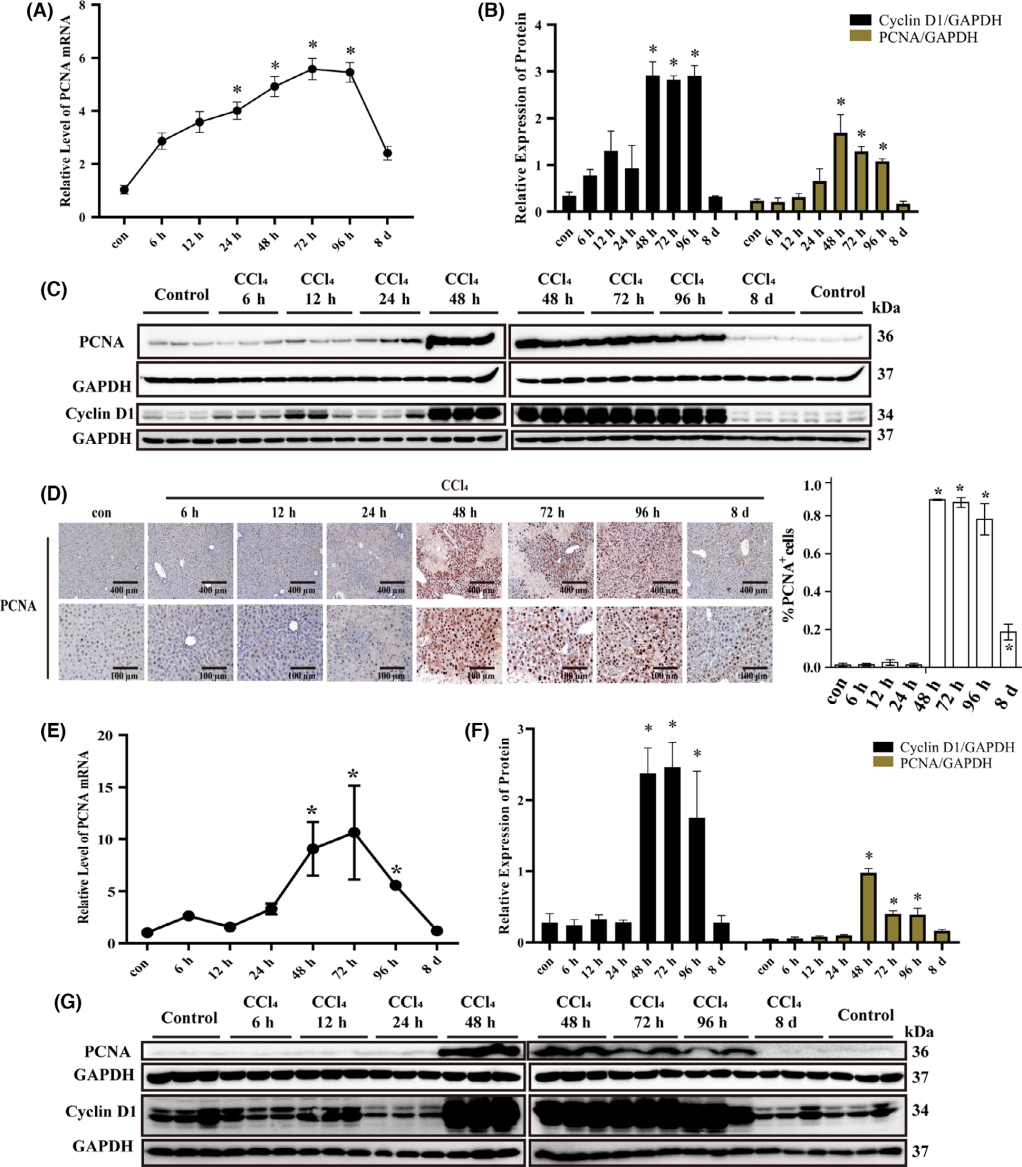
Expression of proliferation‐associated genes in CCl_4_‐induced acute liver injury model mice. RNA levels of Pcna detected using qRT‐PCR in liver tissue (A) and hepatocytes (E); protein level of PCNA and cyclin D1 detected using western blot in liver tissue (B‐C) and hepatocytes (F, G), GAPDH was used as an internal control; (D) expression of PCNA detected in liver tissue using IHC. Scale bar, 400 μm for 10× and 100 μm for 40×. The data were expressed as the mean ± SEM for at least triplicate experiments. *P* values were analyzed by Student’s *t*‐test. *P < 0.05 vs control.

The liver exhibits significant regenerative capacity when injured. PCNA and cyclin D1 play a critical role in DNA synthesis, cell proliferation, and cell cycle regulation, which reflects cell proliferative activities [23]. An increased expression of PCNA and cyclin D1, as shown by the enhancement of mRNA and protein level, was observed at 48 h and maintained for 96 h in CCl_4_‐injured livers, compared with the control (Fig. 4A–C). Further, PCNA and cyclin D1 were expressed in injured primary hepatocytes during 48–96 h, consistent with the results observed in liver tissues (Fig. 4D–G). In summary, our results indicated that considerable liver regeneration occurred in the recovery phase.

### Expression profiles of cytokines in D‐gal/LPS‐induced liver injury

**Fig. 5:**
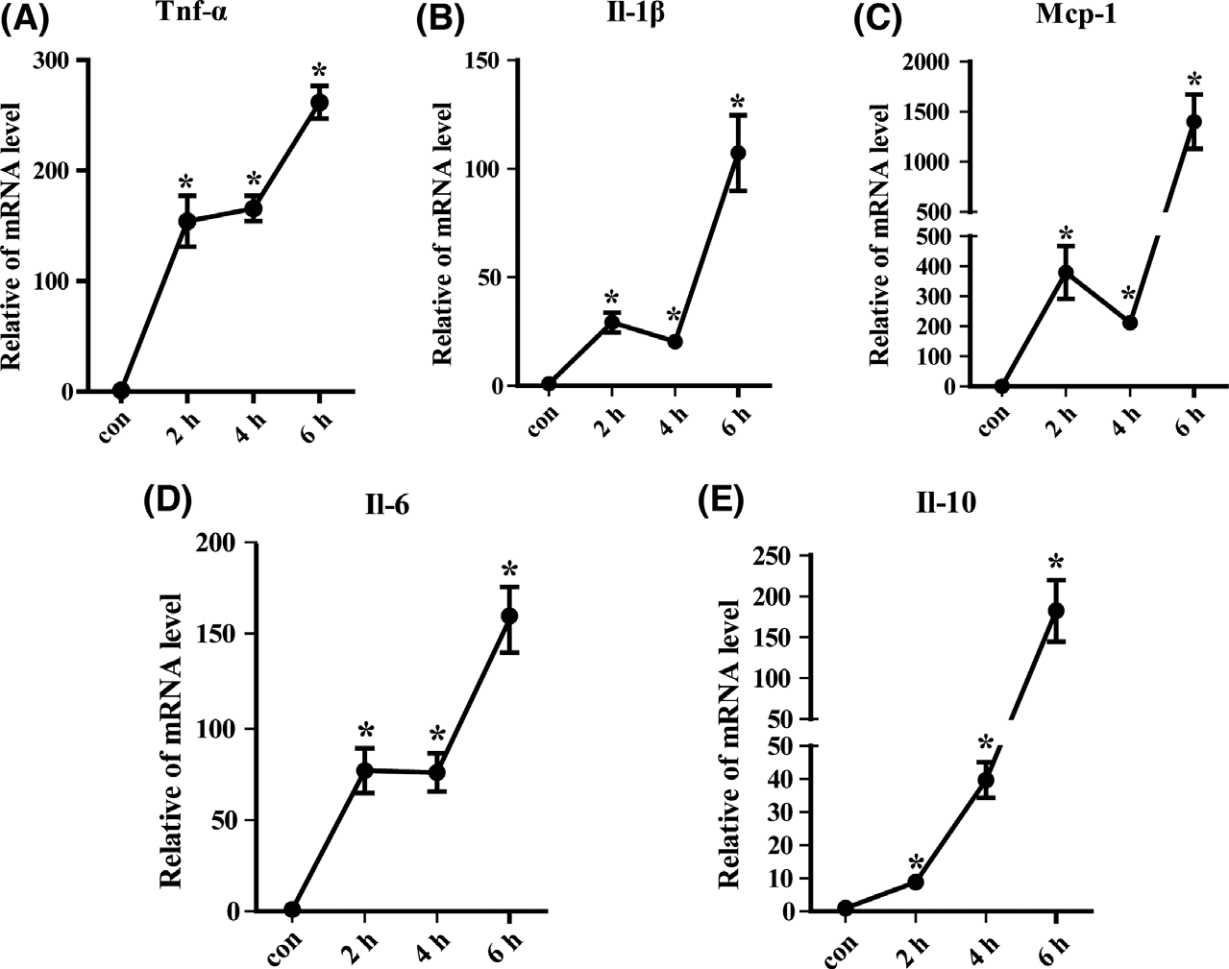
Cytokine expression in D‐gal/LPS‐induced acute liver injury model mice. The expression of inflammation‐related genes TNF‐α (A), IL‐1β (B), Mcp‐1 (C), IL‐10 (D), and IL‐6 (E) was evaluated by qRT‐PCR. The data were expressed as the mean ± SEM for at least triplicate experiments. *P* values were analyzed by Student’s *t*‐test. *P < 0.05 vs control.

Detection of TNF‐α, IL‐1β, IL‐6, Mcp‐1, and IL‐10 showed a significant overexpression 2 h after intraperitoneal injection and peaking at 6 h in the D‐gal/LPS‐induced liver injury compared with the control (Fig 5A–E). These results suggest that coadministration of D‐gal and LPS in mice effectively induced inflammatory response that evidently occurred 6 h following drug injection.

### Dynamics of cell death patterns in D‐gal/LPS‐induced liver injury

**Fig. 6:**
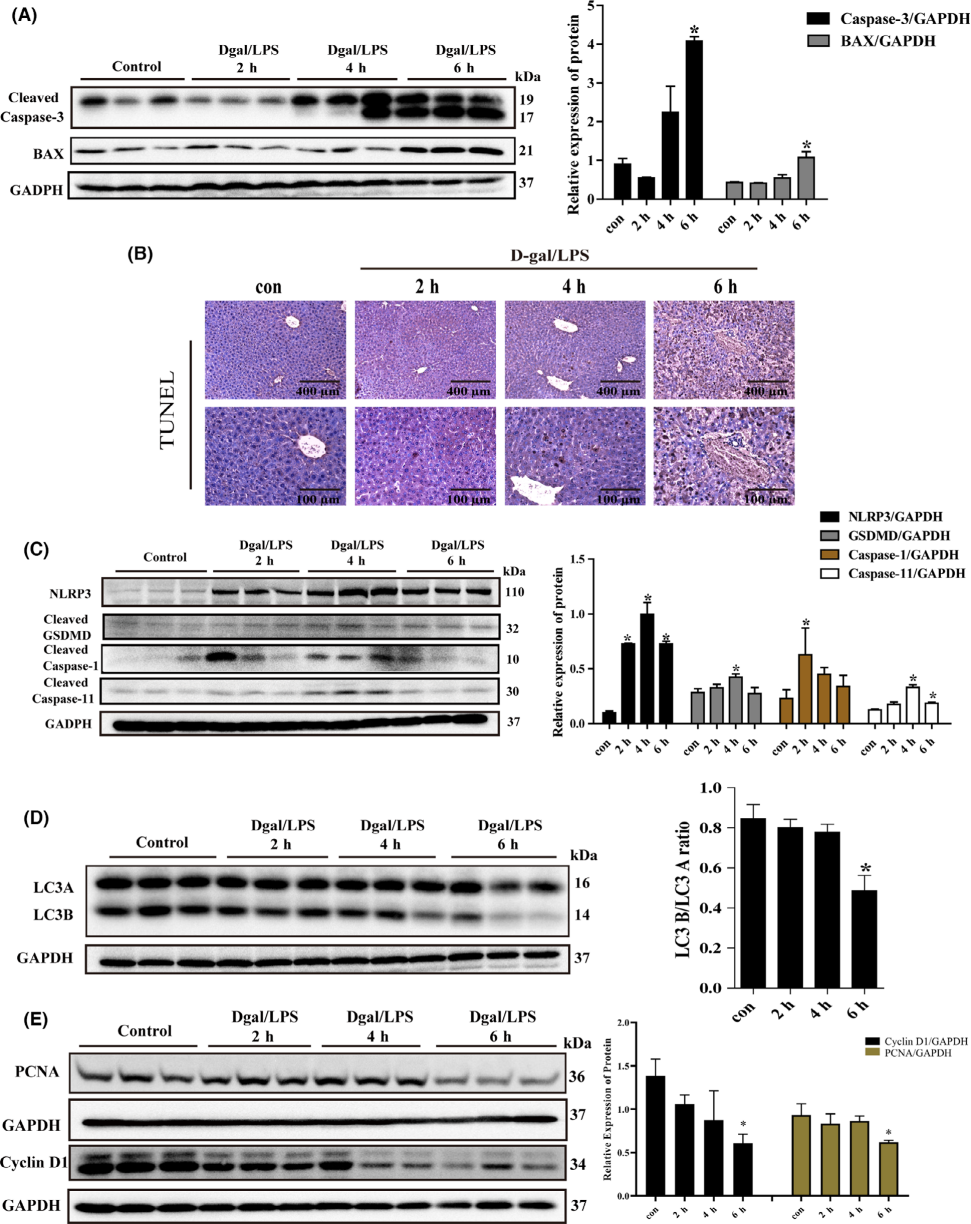
Expression of apoptosis, pyroptosis, autophagy, and proliferation in liver tissues of mice in D‐gal/LPS‐induced acute liver injury model mice. (A) The protein level of apoptosis genes in liver tissues was determined by western blot; (B) TUNEL detection of apoptotic gene expression in liver tissue; (C) the protein level of pyroptosis genes in liver tissue determined by western blot; (D) the protein level of autophagy‐related gene in liver tissue determined by western blot; (E) the protein level of regeneration‐related genes in liver tissue determined by western blot. GAPDH was used as an internal control. Scale bar, 400 μm for 10× and 100 μm for 40×. The data were expressed as the mean ± SEM for at least triplicate experiments. *P* values were analyzed by Student’s *t*‐test. *P < 0.05 vs control.

Cleaved caspase‐3 and BAX were overexpressed at 4 h, reaching peaks at 6 h (Fig. 6A). In addition, TUNEL staining suggested no significant change at 2 and 4 h, but increased significantly at 6 h (Fig. 6B). Taken together, our results indicated that apoptosis was significantly observed at 6 h. The expression of NLRP3 and cleaved caspase‐1 was increased at 2 h, while the expression of GSDMD‐N and cleaved caspase‐11 was increased at 4 h (Fig. 6C). This suggested that in the process of D‐gal/LPS‐induced acute liver injury, pyroptosis occurred in early stage. Additionally, the ratio of LC3‐B to LC3‐A decreased at 6 h after liver injury, indicating decreased autophagy precedes severe liver injury (Fig. 6D). However, the expression of necroptosis‐associated proteins MLKL and ferroptosis‐associated protein GPX4 had no significant change in the development of D‐gal/LPS‐induced acute liver injury (Fig. S2A).

### Dynamics of liver regeneration in D‐gal/LPS‐induced liver injury

The mRNA level of Pcna detected using qRT‐PCR was not altered during 2–6 h in D‐gal/LPS‐injured mice compared with the control group (Fig. S2B). However, the PCNA and cyclin D1 protein detected by western blot were significantly decreased at 6 h (Fig. 6E). These results indicated that liver regeneration was inhibited after severe acute liver damage.

## Discussion

The animal models exposed to CCl_4_ and D‐gal/LPS have been extensively applied for investigating the underlying mechanisms of acute liver injury. CCl_4_ is a widely used chemical toxicant and causes severe liver tissue damage by the cytochrome P450 enzymes to form reactive intermediates, such as trichloromethyl‐free radicals and peroxyl radical, which results in lipid peroxidation and hepatocyte necrosis [24, 25, 26]. Lipopolysaccharide (LPS), also known as endotoxin, is a major component of the outer membrane of Gram‐negative bacteria, which induces a strong inflammatory response [27, 28]. D‐gal is a toxin that depletes uracil nucleotides and triggers apoptosis and necrosis of hepatocytes to induce liver injury, which accompanied by LPS could further aggravate liver injury [29, 30]. If the causative agent persists, it can progress to chronic liver injury. Knockout of HMGB1 has no effect on fibrosis, regeneration, and inflammation in chronic liver injury, but decreases the tumor aggressive markers and hepatocarcinogenesis [31]. By creating two mouse acute liver injury models, the present study investigated the dynamic change of cell death patterns and regeneration during the different stage of liver injury.

Acute liver injury in mice was induced by CCl_4_ or D‐gal/LPS, and histological staining together with serum enzymes demonstrated the models were successfully constructed. Liver damage was most severe 24 and 6 h after CCl_4_ and D‐gal/LPS treatments, respectively. Also, pathological manifestations showed a large area of hepatocyte necrosis, destruction of the hepatic lobular structure, and inflammatory cell infiltration. These results were consistent with those of previous studies that reported most severe liver injury at 24 h after CCl_4_ induction, showing large areas of cell necrosis, loss of hepatic structures, and perivascular inflammatory cell infiltration [24]. The report that the most significant histological grade of liver injury in mice occurred 6 h after D‐gal/LPS induction [32] could be reproduced by our study.

Increased expression of caspase‐1/11, GSDMD‐N, and IL‐1β was observed in the liver tissue of the CCl_4_ group during 6–12 h and the D‐gal/LPS group during 2–4 h, indicating that pyroptosis had occurred in the early stage. In addition, our data revealed that the pyroptosis‐associated gene showed no obvious changes until 24 h in injured primary hepatocytes, but significantly increased at 6 h in KCs, suggesting that KCs, instead of hepatocytes, were the main cells involved in pyroptosis in the early stage of acute liver injury. KCs, as important nonparenchymal cells in the liver, are the liver’s first line of defense after injury, and are activated by pathogen‐ and damage‐associated molecular patterns, which lead to the recruitment of innate effector cells to the injured liver [33, 34]. In our study in recent years, there has been increasing evidence of pyroptosis in KCs during liver damage; for instance, recent study reported that rare earth oxide induced NLRP3 inflammasome activation, caspase‐1 activation, and pyroptosis in KCs. Pyroptosis was accompanied by cell swelling and IL‐1β release, which could be reversed by knockdown of GSDMD [35]. Further, our previous research demonstrated that chronic liver injury is related to inflammation and cell pyroptosis. CCl_4_ and bile duct ligation (BDL) treatment significantly increased protein levels of NLRP3, caspase‐1, and GSDMD‐N, while silencing lnc‐Lfar1 ameliorated CCl_4_‐ and BDL‐induced NLRP3 inflammasome‐mediated pyroptosis [36]. Therefore, we suggested that pyroptosis was triggered in KCs and the cells subsequently released inflammatory mediators in the early stages of liver injury, leading to hepatocyte injury and death. In addition, several studies have shown that autophagy exists in mice with acute liver injury. Autophagy, as a cellular protective mechanism, can reduce hepatocyte necrosis, while inhibition of autophagy can promote hepatocyte necrosis [37, 38]. In this study, we found that expression of autophagy‐related gene LC3‐B/LC3‐A was significantly upregulated in the CCl_4_ model, but not changed in the D‐gal/LPS model. This indicated that autophagy may possibly serve as a compensatory mechanism during early liver injury in the CCl_4_ model, and induction of autophagy may improve hepatocyte survival by providing energy against an adverse environment.

Liver damage was most severe in the CCl_4_‐treated group at 24 h and in the D‐gal/LPS‐treated group at 6 h, which was considered the progressive stage of liver damage. Morphological results revealed a smaller necrotic area of liver cells in the CCl_4_ group than in the D‐gal/LPS group, and less congestion and hemorrhage in the necrotic area, and the survival hepatocytes could be observed. In order to further explore the differential mechanisms of liver injury, we conducted a systematic study of cytokines, cell death, and regeneration in these two models. After liver injury, KCs are activated and cascade to release cytokines and inflammatory mediators, in which TNF‐α not only has a strong proinflammatory effect but also exhibits strong proapoptotic activity [39, 40]. Apoptosis is also an important feature of D‐gal/LPS‐induced acute liver injury [41]. A high level of TNF‐α induces massive apoptosis of hepatocytes through a death receptor‐dependent pathway, which leads to severe liver injury [42]. It has been reported that recombinant human augmenter of liver regeneration (rALR) treatment decreased the chemokine expression, oxidative stress, and apoptosis, resulting in less inflammatory cell infiltration and reduced hepatic tissue damage after hepatic ischemia/reperfusion injury [43]. In our study, we found that apoptosis was increased and the expression of cytokines was significantly upregulated after D‐gal/LPS treatment for 6 h, indicating the inflammatory response was strong. In addition, the downregulated expression of LC3‐B, PCNA, and cyclin D1 suggested that liver autophagy and regeneration were inhibited during the progression of severe liver injury. The expression of autophagy‐related markers was significantly upregulated in the CCl_4_ group after 24 h, whereas the expression of cytokines such as TNF‐α and IL‐1β was only mildly elevated, with much less intense inflammatory response than the D‐gal/LPS group. We believe that the extent of liver damage in the D‐gal/LPS group was significantly more severe and the prognosis was worse than the CCl_4_ group because D‐gal/LPS treatment triggered a strong cytokine storm, resulting in severe hepatocyte necrosis, significant apoptosis, and inhibited autophagy and regeneration. Comparatively, cytokine levels fluctuated after CCl_4_ treatment, but the increase was relatively low, and cell autophagy was active. Therefore, the level of cytokines and cell death detection are important references for the prognostic evaluation of acute liver injury.

Lastly, we investigated changes in liver regeneration, inflammatory factors, and apoptosis in the recovery phase of liver injury (CCl_4_ group, from 48 h to 8 days). TNF‐α, IL‐6, Mcp‐1, caspase‐3, and BAX were upregulated, while expression of PCNA and cyclin D1 in liver tissue and hepatocytes increased significantly, indicating that proliferation and repair of hepatocytes played an important role in liver regeneration. At this stage, compensatory hepatocyte proliferation begins, and dead cells are replaced by new cells, contributing to liver regeneration and recovery. In the initial stage of liver regeneration, TNF‐α and IL‐6 are important regulatory factors, which can activate resting liver cells to enter the cell cycle to replicate [44]. Regeneration of macrophages mediated by Mcp‐1 is another important mechanism of liver regeneration [45]. In addition, apoptosis is closely related to liver regeneration. Apoptotic cells can release signal molecules such as cytokines, growth factors, and prostaglandins to promote regeneration [46]. Knockout of lymphotoxin‐β receptor led to prolonged and exacerbated liver pathology with larger necrotic areas and increased apoptotic cell death, which result in aggravation of liver injury and delay of liver regeneration [47]. Additionally, different concentrations of CXC chemokines regulate the hepatic proliferation and subsequent regeneration [48]. Thus, CCl_4_ model mice recovered from liver injury upon initiation of a strong hepatic regenerative response, while liver regeneration failed in the D‐gal/LPS group, in which the continued progress of liver damage eventually led to significant mortality.

**Fig. 7:**
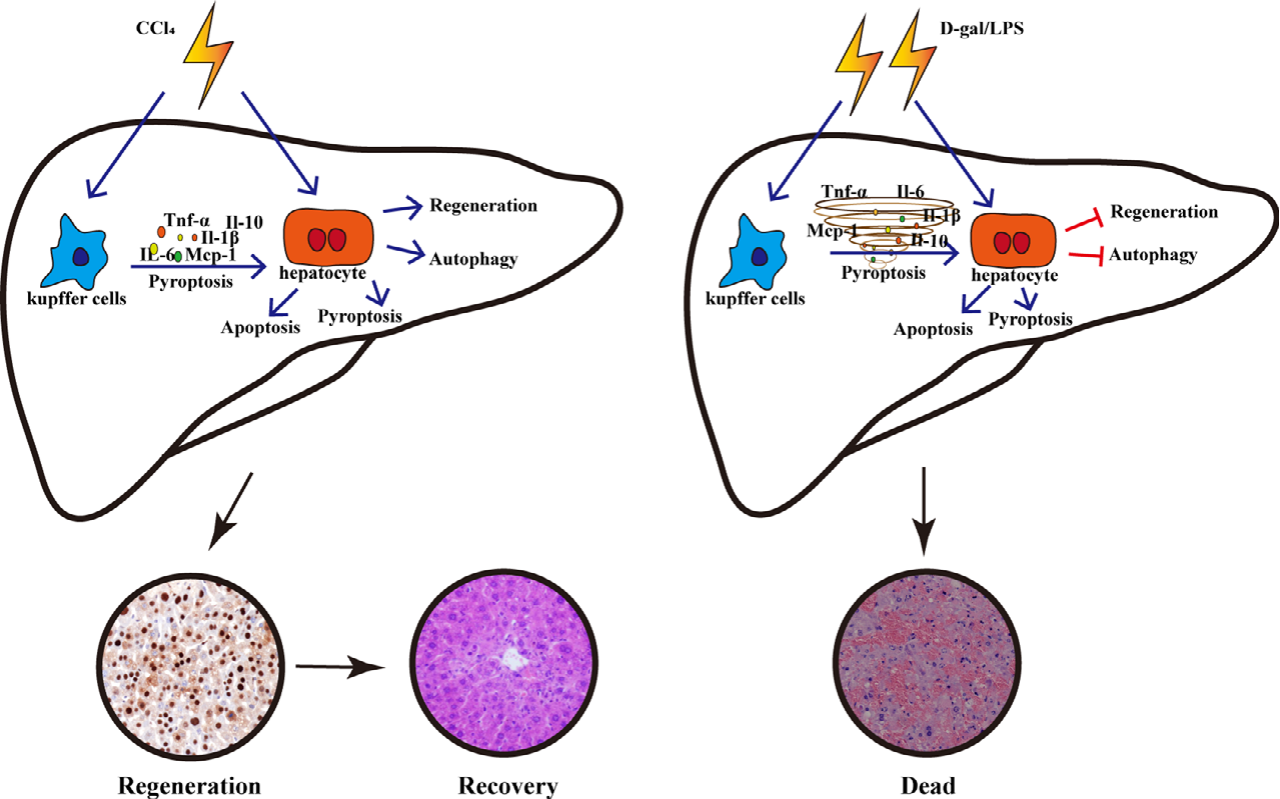
Schematic diagram illustrating the progress of CCl_4_‐ and D‐gal/LPS‐induced acute liver injury model mice.

The results of this study demonstrated that three phases in the development of CCl_4_‐induced acute liver injury were considered in this model, and the change in cytokines showed double‐peak fluctuations, both of which were not described previously by other studies. Additionally, cell apoptosis and pyroptosis persisted throughout all phases of acute liver injury, significant cell autophagy occurred in the early phase, and sufficient and timely hepatocyte regeneration occurred in the recovery phase, contributing to relatively mild liver injury and favorable prognosis. However, only the early and progressive phases were observed in D‐gal/LPS‐induced acute liver injury. With liver autophagy and regeneration were significantly inhibited, pyroptosis occurred in the early phase and significant apoptosis occurred in the progressive phase, accompanied by a strong cytokine storm resulting in severe liver injury with high mortality (Fig. 7). In this study, we have detected different manners of cell death including apoptosis, pyroptosis, ferroptosis, necroptosis, and autophagy during acute liver injury in the same animal model and found that distinct cell death occurred at different phases of injury, all of which have not been demonstrated previously. In conclusion, our work revealed variable manners and dynamics of cell death and regeneration, which lead to different consequences in the mild and severe acute liver injury, providing a helpful reference for clinical therapy and prognosis.

## Conflict of interest

The authors declare no conflict of interest.

## Author contributions

WH and TH conceived and designed the studies. SS, YZ, GL, ZY, and YC performed the majority of the experiments. LZ, XH, ZS, HC, and XS helped to perform the histological experiments. SS, WH, TH, and KZ analyzed and interpreted the data. SS and YZ drafted the manuscript. WH and TH served as the project leader and extensively and critically revised this manuscript. All authors reviewed and commented on the manuscript and approved the final version.

## Supporting information


**Fig. S1**. The protein level of pyroptosis‐ and ferroptosis‐associated genes in CCl_4_‐induced acute liver injury.
**Fig. S2**. The expression of PCNA and ferroptosis‐ associated genes in D‐gal/LPS‐induced mice injured liver.

## Data Availability

The data that support the findings of this study are available from the corresponding author. Correspondence and requests for materials should be addressed to TH (hantaomd@126.com).
