# Supplementary material for: The dynamics of cell death patterns and regeneration during acute liver injury in mice
Source: FEBS Open Bio. 2022 Mar 5;12(5):1061–74. doi: 10.1002/2211-5463.13383 (PMC9063440; doi:10.1002/2211-5463.13383)
Supplement: Supplementary file 1 — Fig. S1. The protein level of pyroptosis‐ and ferroptosis‐associated genes in CCl4‐induced acute liver injury. Fig. S2. The expression of PCNA and ferroptosis‐ associated genes in D‐gal/LPS‐induced mice injured liver. [file FEB4-12-1061-s001.docx]

**
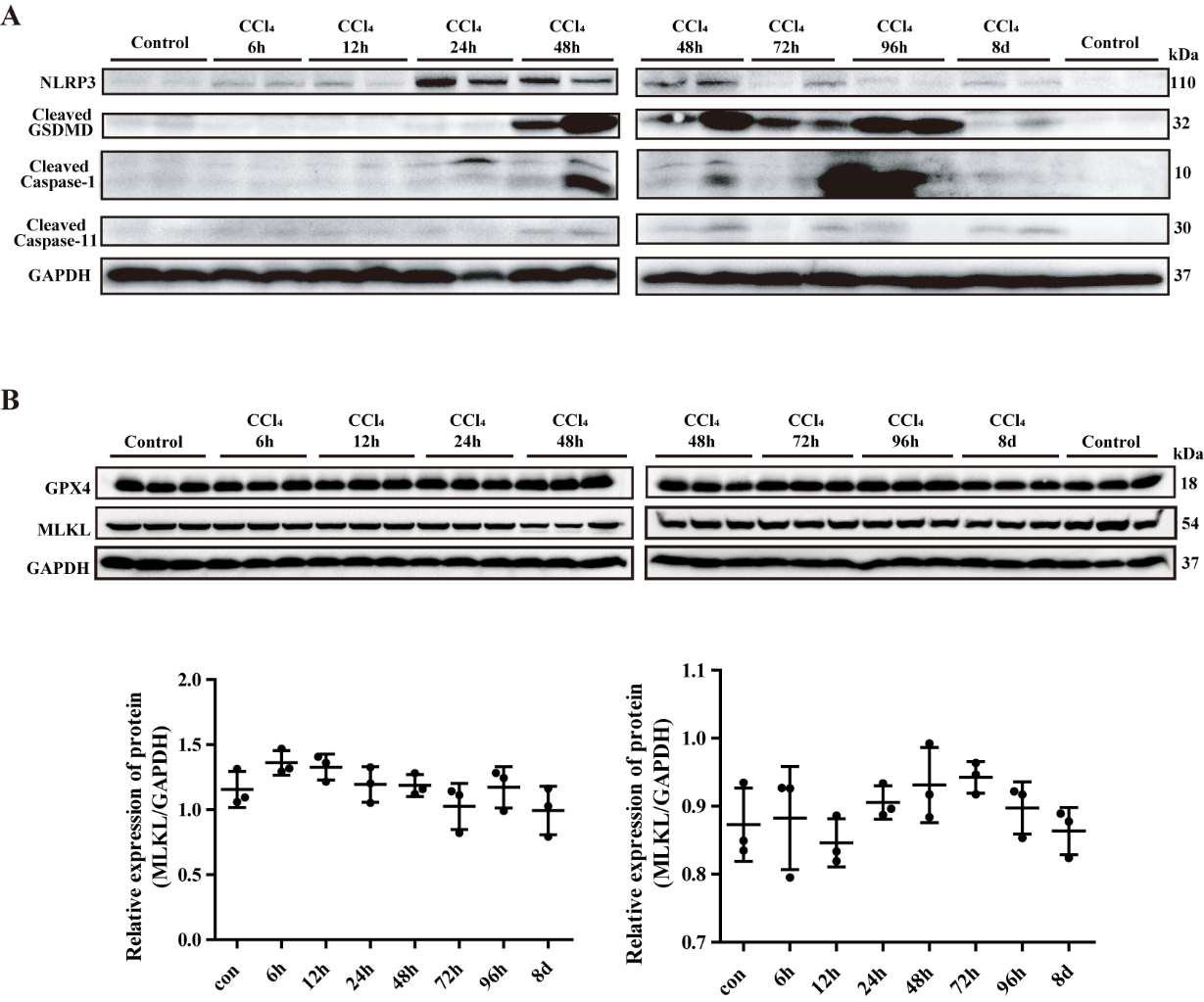
**

**Supplementary Figure. S1**

Mice were injected with either CCl_4_ (olive oil as control) to induce acute liver injury and were sacrificed at different time points. (A) The protein level of pyroptosis genes in injured primary hepatocytes determined by western blot; (B) The protein level of MLKL and GPX4 in liver tissue detected by western blot. GAPDH was used as an internal control.


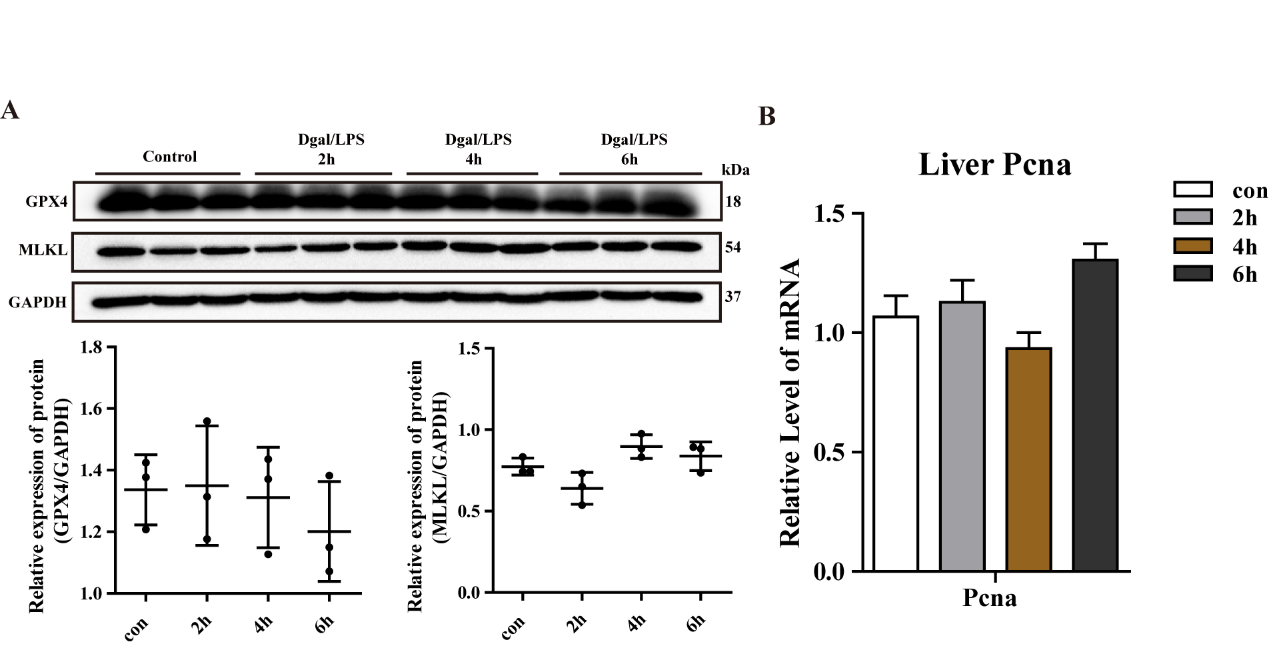


**Supplementary Figure. S2**

Mice were injected with D-gal/LPS (saline as control) to induce acute liver injury and were sacrificed at different time points. (A) The protein level of MLKL and GPX4 in liver tissue detected by western blot. GAPDH was used as an internal control; (B) The expression of Pcna detected in liver tissue using qRT-PCR. The data are presented as the mean ± SEM for at least triplicate experiments.
